# Special Issue: Advanced Nanocomposite Materials Based on Graphene Oxide/Reduced Graphene Oxide: Potential Applications and Perspectives

**DOI:** 10.3390/ma15248983

**Published:** 2022-12-15

**Authors:** Mariano Palomba, Angela Longo

**Affiliations:** Institute for Polymers, Composites, and Biomaterials, National Research Council, 80055 Portici, Italy

In recent years, graphene oxide (GO) and reduced graphene oxide (r-GO) have received much attention as precursors of graphene-like 2D nanomaterials. GO consists of a layered material based on a carbon skeleton functionalized by different oxygen-containing groups (typically with a C/O atomic ratio of less than 3), and r-GO is obtained via the almost complete removal of oxygen functional groups [1,2,3]. These materials have unique intrinsic physical and chemical properties, including a large surface area, functional groups, good conductivity, and good biocompatibility. For this reason, many experimental studies have been conducted to improve the preparation methods of GO and r-GO, and to analyze their possible applications [4,5]. Recently, a growing number of studies have been published concerning the preparation and characterization of new nanocomposites, which integrate GO and/or r-GO (GO/r-GO) with polymers [6], and inorganic nanoparticles (metal, metal oxide, etc.) [7].

For example, GO/r-GO-based polymer nanocomposites are receiving remarkable interest due to their excellent mechanical, thermal, and electrical properties. GO/r-GO can be used as nanometric fillers embedded in a polymeric matrix to enhance the structural, morphological, and functional properties of the composite material [3]. In addition, these materials are suitable for electronic and energy storage applications in the form of polymer–graphene composites [8]. Furthermore, nanocomposites based on GO/r-GO and inorganic nanoparticles such as Au, Ag, and Pt have attracted much attention due to their applications as catalysts, photocatalysts, electrodes, sensors, substrates for surface-enhanced Raman spectroscopy, and in biomedicine [7].

This Special Issue contains unique articles and reviews that reflect the current state-of-the-art, focusing on the performance peculiarities of nanocomposite materials based on GO and/or r-GO in specific fields of application.

Jiang and co-workers studied the in situ growth of Fe_3_O_4_ nanoparticles as homogeneous clusters on reduced graphene oxide [8]. In fact, the results showed that Fe_3_O_4_ nanoparticles, anchored on r-GO surfaces, formed nanoclusters without aggregation, and they expanded the interlayer spacing between r-GO sheets. The improved electrochemical properties of the Fe_3_O_4_/r-GO nanocomposite compared with pure Fe_3_O_4_ can be attributed to high electron transport, increased interfaces, and positive synergistic effects between Fe_3_O_4_ and r-GO. These nanocomposites represent an effective strategy to develop new advanced supercapacitor electrodes for energy storage devices [8].

Ullah et al. described the nanocomposite synthesis of magnetic nanoparticle (MNPs) and r-GO in polymethylmethacrylate (PMMA) [9]. The combination of the high performance of PMMA (due to its mechanical and physical properties) with active adsorbents, such as GO magnetized using FeCl_3_ and FeSO_4_ salts, was proven to be an interesting approach for the removal of hazardous Cr(VI) from tannery wastewater through batch- and continuous-mode adsorption [9].

Regarding new materials for storing solar energy, which have received a great deal of attention, Yang et al. [10] studied a photothermal conversion material obtained by attaching trifluoromethylated azobenzene (AzoF) to reduce GO. This system exhibited remarkable energy storage performance, as well as an excellent storage life span, and it is equipped with the ability to release heat at low temperatures [10].

Due to the SARS-CoV-2 pandemic, there has been an increase in the search for affordable healthcare devices for mass testing and rapid diagnosis. In this context, Zaccariotto et al. [11] described a new methodology for SARS-CoV-2 detection based on an impedimetric immunosensor developed using the advantageous immobilization of antibodies in the r-GO. An electrochemical immunoassay was considered for the detection of the SARS-CoV-2 spike protein RBD using a impedimetric immunosensor and redox couple ([(Fe(CN)_6_)]^3−/4−^) as a probe [11].

This Special Issue also presents three interesting reviews that could act as good starting points for the development of innovative materials based on GO, using green processes characterized by good feasibility, cost-effectiveness, and sustainability.

Leve et al. [12] report on several literature results pertaining to the promising functionalization of GO and r-GO surfaces with metal oxide, for enhanced performance in selectivity and sensitivity in gas sensing. In this review, the functionalization of graphene, the synthesis of heterostructured nanohybrids, and the assessment of their collaborative performance towards gas-sensing applications are discussed [12].

Wei et al. [13] studied graphene-based composite aerogels (GCAs), which are a solid porous substance formed by graphene or its derivatives, graphene oxide (GO) and reduced graphene oxide (rGO), with inorganic materials and polymers. This review demonstrates the super-high adsorption, separation, electrical properties, and sensitivity of GCA, which could have great potential for applications in super-strong adsorption and separation materials, long-life fast-charging batteries, and flexible sensing materials [13].

Palomba et al. [14] analyzed papers from the literature concerning the most important approach to GO reduction based on the use of L-ascorbic acid. The results were organized according to two important approaches: reduction in the liquid-phase and in the gel-phase. The achieved r-GO quality enabled its technological exploitation in various forms; for example, it could be used as a coating, self-supported, or embedded in a polymer. Knowledge of all aspects of the synthesis and properties of r-GO obtained using the L-aa reduction technique is critical in bringing this process into mass production [14].

As demonstrated in this Special Issue, the design and development of nanocomposites based on GO/r-GO with tailored properties are essential in order to expand their range of potential applications. We hope this will stimulate further development and new ideas by prompting fruitful discussions between academic and industry experts who work in the field of graphene-related materials.
